# Toward Human Digital Twins for Cybersecurity Simulations on the Metaverse: Ontological and Network Science Approach

**DOI:** 10.2196/33502

**Published:** 2022-04-20

**Authors:** Tam N Nguyen

**Affiliations:** 1 Department of Management Information Systems University of Arizona Tucson, AZ United States

**Keywords:** human behavior modeling, cognitive twins, human digital twins, cybersecurity, cognitive systems, digital twins, Metaverse, artificial intelligence

## Abstract

**Background:**

Cyber defense is reactive and slow. On average, the time-to-remedy is hundreds of times larger than the time-to-compromise. In response, Human Digital Twins (HDTs) offer the capability of running massive simulations across multiple domains on the Metaverse. Simulated results may predict adversaries' behaviors and tactics, leading to more proactive cyber defense strategies. However, current HDTs’ cognitive architectures are underdeveloped for such use.

**Objective:**

This paper aims to make a case for extending the current digital cognitive architectures as the first step toward more robust HDTs that are suitable for realistic Metaverse cybersecurity simulations.

**Methods:**

This study formally documented 108 psychology constructs and thousands of related paths based on 20 time-tested psychology theories, all of which were packaged as Cybonto—a novel ontology. Then, this study applied 20 network science centrality algorithms in ranking the Cybonto psychology constructs by their influences.

**Results:**

Out of 108 psychology constructs, the top 10 are Behavior, Arousal, Goals, Perception, Self-efficacy, Circumstances, Evaluating, Behavior-Controllability, Knowledge, and Intentional Modality. In this list, only Behaviors, Goals, Perception, Evaluating, and Knowledge are parts of existing digital cognitive architectures. Notably, some of the constructs are not explicitly implemented. Early usability tests demonstrate that Cybonto can also be useful for immediate uses such as manual analysis of hackers’ behaviors and automatic analysis of behavioral cybersecurity knowledge texts.

**Conclusions:**

The results call for specific extensions of current digital cognitive architectures such as explicitly implementing more refined structures of Long-term Memory and Perception, placing a stronger focus on noncognitive yet influential constructs such as Arousal, and creating new capabilities for simulating, reasoning about, and selecting circumstances.

## Introduction

### The General Landscape

Humans are well recognized as the weakest link in the cybersecurity defense chain [1,2]. Insider threat incidents cost both small and large companies billions of dollars annually [3]. Nonetheless, cyber defenders are still reactive and slow. On average, hackers need 15 hours to compromise a system, while defenders need 200 to 300 days to discover a breach [2]. Meanwhile, the cybersecurity threat landscape keeps expanding. Cyber defenders respond by enlisting interdisciplinary knowledge from numerous fields such as mathematics, psychology, and criminology [2,4-6]. In such a climate, Digital Twins (DTs) and Human Digital Twins (HDTs) offer the capability of running simulations across multiple knowledge domains on the Metaverse to improve proactive cyber defense strategies.

DTs are computational models of physical systems, including humans. The DT market is rapidly growing at a compound annual rate of 45.4% [7]. Notably, massive DT projects such as the British National Digital Twin [8] are being built. Within the intertwined DT networks, individual smart DTs such as HDTs should be capable of not only executing mimetic behaviors but also having local and global awareness, self-learning, and self-optimizing [7].

HDTs should coexist with other DTs within the paradigm of agent-based modeling and simulation for cybersecurity. Nonhuman DTs can be components of an Information Systems (routers, servers, and Internet of Things systems), while HDTs are the system users, system admins, and malicious actors. Agent-based modeling offers cost-effective, rigorous, and risk-free scenario testing that should inspire more proactive cybersecurity defense strategies. The *Prior Work* section discusses some use cases of HDTs and agent-based modeling in cybersecurity.

Zooming out to a broader perspective, the “Metaverse” is a gigantic, persistent, and unified realm of various virtual environments such as DT networks, social networks, digital publishing networks, virtual 3D networks, cyber-physical infrastructures, cloud infrastructures, and blockchains. Lee et al [9] proposed a “digital twin-native continuum” reflecting three Metaverse development stages. The first stage mainly involves digital twins and the effort of digitalizing the real world. In the next stage, digital twins and other virtual entities form isolated cyber-physical environments that are called “many virtual worlds.” Finally, the many virtual worlds will be connected to form the Metaverse. The paper focuses on this vision for the Metaverse in which large-scale simulations can be collaboratively done by massive networks of interconnected DTs.

### Backgrounds on HDTs

The concept of HDTs previously appeared in human-computer interaction studies. In comparison with traditional models, HDTs for the Metaverse have broader scopes with emphasis on both behavioral and cognitive activities. The work of Somers et al [10] is an excellent example in which HDT acts as a sensible personal assistant in organizing social events. Notably, the HDT did not explicitly ask potential event participants for their preferences. Instead, it observed the people’s social dimensions and then modeled the cognitive processes underlying an expert event planner’s decision.

Such a continuous process of dynamic knowledge acquisition and utilization was described by Zhang et al [11] as HDTs’ self-awareness involving numerous feedback loops. Well-designed ontologies are essential for those information exchange loops [12,13]. Among ontologies, reference ontologies are supposed to be much more canonical and reusable than application ontologies [14].

### Backgrounds on Cognitive Frameworks

Cognitive frameworks are essential for building HDTs’ cognitive features. ACT-R [15] is representative of the psychological modeling group with Clarion and Epic as other members. SOAR [16] is representative of the agent functionality–focused group, which also includes Sigma, Lida, Icarus, and Companions. ACT-R and SOAR differ on architectural constraints, memory retrieval, conflict resolution strategies, and exhaustive processing [17]. ACT-R sequential architecture forces developers to watch out for bottlenecks, while SOAR’s parallel architecture is more relaxed [17]. ACT-R provides two options for resolving conflicts, while SOAR offers none.

Both SOAR and ACT-R share the same general cognitive cycle and common architectural modules such as perception, short-term memory, declarative learning, declarative long-term memory, procedural long-term memory, procedural learning, action selection, and action. While ACT-R, SOAR, and other cognitive systems rely on the symbolic input or output and rule database, their symbols may contain statistical metadata, and their architectures allow for the integration of deep learning systems.

### Backgrounds on Cybersecurity Ontologies

Ontologies are essential for HDTs’ feedback loop communications, symbolic operations, the building of a knowledge base, and explainability. Ontologies can be manually built from scratch [18,19] or be automatically extracted [20,21]. DOLCE [22] vs Basic Formal Ontology (BFO) [14] highlights the importance of ontological commitments by choosing a top-level ontology. DOLCE top-level ontology is grounded in natural language, while BFO top-level ontology is grounded in the real world [23]. Because objects can be conceptual or actual in a language-based ontology, there is always a risk of one actual object being recognized as two or more different conceptual objects.

Oltramari et al [24] introduced Cratelo, which is based on DOLCE. The ontology’s human behavioral structures are confined within the cyber operation scope. Costa et al [25] used the natural language processing approach in building their Insider Threat Indicator Ontology. The ontology inherited considerable amounts of language ambiguity and did not support the identification of deeper behavioral structures. In 2019, Greitzer et al [26] built upon their 2016’s work and introduced the Sociotechnical and Organizational Factors for Insider Threat (SOFIT). Owing to the absence of a top-level ontology and the behavioral language that leans heavily toward organizational insider threat activities, SOFIT is an application ontology rather than a reference ontology. Greitzer et al [26] also admitted that ontology validation exercises only covered 10% of the ontology.

Meanwhile, Donalds and Osei-Bryson [27] reported that cybersecurity ontologies have been insufficient owing to fragmentation, incompatibility, and inconsistent use of terminologies. The team proposed a cybercrime classification ontology structured around attack events [27]. While the ontology provides a holistic, multi-perspective view regarding cybercrime attacks, its behavioral components are limited and lack theoretical grounding.

### Open Problems

While massive DT projects are underway, digital cognitive twin development is pale in comparison, and HDT for cybersecurity is underdeveloped. This paper examined both ACT-R– and SOAR-published research repositories and found no cybersecurity-dedicated track with topics such as cybersecurity, web-based ethical decisions, cyber criminology, or cyberattack or defense simulations. Recommended explorative questions are as follows: (1) What types of HDT (malicious hackers, groups as single HDT, and defenders) should be built? (2) What will HDT for cybersecurity feedback loops look like? (3) How will existing cognitive architectures be extended to best facilitate those feedback loops? (4) What shall we learn from our continuous observation of those HDTs on the Metaverse?

Current cybersecurity-related autonomous agents focus on narrow tasks and are far from the HDTs that can automatically interact with other DTs while building up their own awareness. For one reason, existing cognitive architectures do not provide enough granularity. This leads to further problems with multimodal understanding and meta-cognition. For example, current long-term memory architecture can be further divided into experiences and beliefs. It is possible for two persons sharing a strong belief to have different interpretations of the same data (difference experiences). Additionally, processing big chunks of data owing to a lack of granularity may lead to cognitive bottlenecks at system levels. Deciding which chunks of data to be loaded, excluded, or be permanently erased from memory remains a challenge.

Finally, we do not have a reference ontology for documenting and sharing behavioral cybersecurity knowledge among humans and DTs. Existing cybersecurity ontologies that have behavioral components are mostly application ontologies with none or weak ontological commitments. Such ontologies will not fit for use in massive networks of DTs on the Metaverse.

Therefore, this paper aims to make a case for extending the current digital cognitive architectures as the first step toward more robust HDTs that are suitable for realistic Metaverse cybersecurity simulations. This paper proposes the Cybonto Conceptual Framework—a grounded and scoped vision on how interconnected DTs and HDTs on a Metaverse may predict real-world behaviors and tactics of hackers. Specifically, the paper unified 20 most cybersecurity-relevant finalists from a knowledge body of over seventy behavioral psychology theories. The theory-informed knowledge and other cybersecurity constructs were then encoded as the novel Cybonto ontology, which sits at the framework’s core and is the paper’s key contribution.

## Methods

### Identifying Relevant Theories

In total, 50 candidate theories were selected from the behavioral or cognitive psychology body of knowledge with more than 70 theories. Each theory was ranked in accordance with its ability to generate research, relevancy to cybersecurity or criminology, and consistency. Table 1 presents the top 25 theories.

For each theory’s original peer-reviewed paper, the total number of citations and the publication year were extracted and used to calculate the citations per year value. The “Google Scholar Results” value (value A) is the total number of Google Scholar search results of the search query (query A) containing the quoted theory’s name and its founder’s last name. The keyword “cybersecurity” was added to the previous search query to form a new query (query B) and get a new search result value (value B). Value B was divided by value A to form the “CySec Density” metric. “CySec impressions“ is the total number of cybersecurity relevant papers within the top 10 papers automatically ranked and displayed by Google Scholar after performing query B. Similarly, “Criminology Impressions” is the result of repeating the same steps for calculating “CySec Impressions” but with the “criminology” keyword instead. All values were normalized into a range from 0 to 10. The final ranking score is the average of “Fitted citations per year,” “CySec Impressions,” “Criminology Impressions,” and “CySec Density Fitted.”

**Table 1:** Top 25 cybersecurity applicable behavioral theories.

Theory name	Google Scholar results, n	CySec Impressions	CySec Density Fitted	Criminology impressions	Fitted citations per year	Final score
Protection Motivation Theory [28]	10,500	10	9	7	0	6.5
Prospect Theory [29]	66,200	8	1	6	10	6.3
General Theory of Crime [30]	13,500	9	1	10	1	5.3
Self-Efficacy Theory [31]	212,000	9	0	6	5	5
Social Norms Theory [32]	47,400	7	9	2	0	4.5
Affective Events Theory [33]	6880	10	1	6	0	4.3
Differential Association Theory [34]	10,700	9	1	7	0	4.3
Extended Parallel Processing Model [35]	412	7	4	6	0	4.3
Focus Theory of Normative Conduct [36]	6220	6	10	1	0	4.3
Containment Theory [37]	2240	9	1	6	0	4
Theory of Planned Behavior [38]	85,800	9	1	3	3	4
Social Identity Theory [39]	66,200	7	0	7	1	3.8
Goal Setting Theory [40]	51,700	6	1	7	1	3.8
Transtheoretical Model of Behaviour Change [41]	35,900	6	0	7	0	3.3
Self-Determination Theory [42]	165,000	8	0	4	0	3
Operant Learning Theory [43]	40,500	7	1	4	0	3
Social Cognitive Theory [44]	162,000	8	0	3	1	3
Change Theory [45]	54,700	8	0	2	0	2.5
Precaution Adoption Process Approach [46]	2590	6	1	3	0	2.5
Diffusion of Innovations [47]	96,700	4	1	3	2	2.5
Control Theory [48]	11,500	6	1	1	0	2
Risk as Feelings Theory [49]	550	5	2	1	0	2
Social Learning Theory [50]	145,000	2	0	6	0	2
Norm Activation Theory [51]	4610	5	1	1	1	2
Technology Acceptance Model [52]	48,100	2	3	1	2	2

A full table with links to Google Scholar queries, descriptions of Cybonto in RDF store, the Neo4J relational database, theory ranking details, and other documentation is available at Cybonto-1.0 GitHub repository [53].

### Ontology Designing

Cybonto elected the BFO as its top-level ontology from more than 30 candidates. BFO [14] is the only top-level ontology that adopts materialism, commits to actual-world possibilia, and has an intensional criterion of identity. The Cybonto Core is grounded further by employing Mental Functioning (MF) as its mid-level ontology. MF follows best practices outlined by the OBO Foundry and aligns with other projects in the Cognitive Atlas—a state-of-the-art collaborative knowledge-base in Cognitive Science [54].

Materialism is the key ontological commitment. It views the world as a collection of materialized objects existing in space and time [23]. Committing to materialism through BFO offers a fundamental distinction in the way Cybonto represents psychological constructs. For centuries, psychological activities were considered abstract particulars that could only be described through languages. This tradition is the reason why most behavioral components in cybersecurity ontologies are language based. Recent breakthroughs in the brain-machine interface such as those of Neuralink [55] enable measurements of brain activities that correspond to certain cognitive constructs. Therefore, it is now possible to ground behavioral or cognitive ontologies in materialism. Cybonto rejects conceptual objects, different linguistic descriptions of the same actual objects, process-based objects, and object labels that cannot be measured in real life.

**Figure 1:**
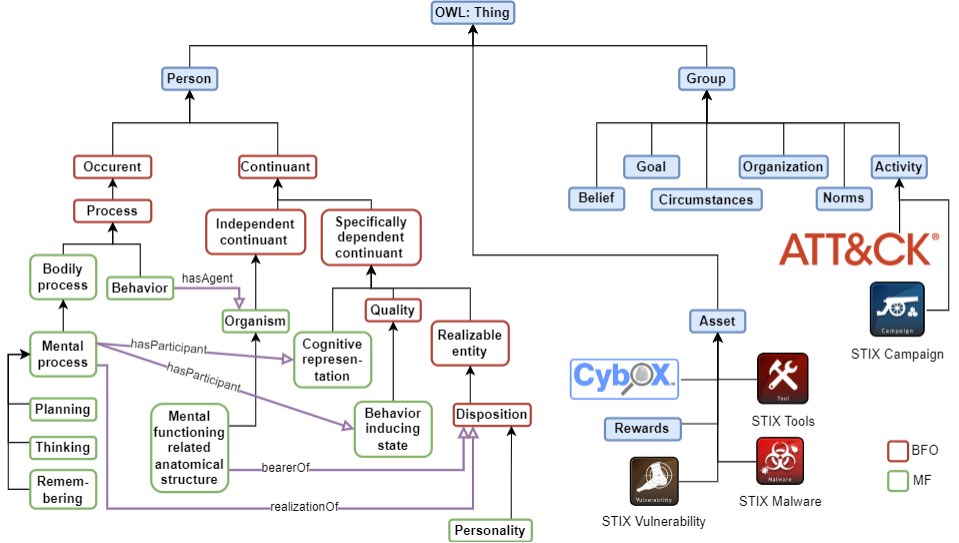
Cybonto's main hierarchies. BFO: Basic Formal Ontology; MF: Mental Functioning.

Figure 1 shows the main hierarchies of Cybonto. The current Cybonto core is based on the top 20 psychology theories. Each chosen one was codified into tuples of (construct, “influence” relationship, and construct). A total of 108 constructs and the relationships among them were put under MF (green), which is covered by BFO (red) under Person. All these constructs form the “Cybonto core.”

Cybonto chooses MITRE’s ATT&CK framework [56] as the main taxonomy for malicious behaviors under both Person and Group classes. The ATT&CK framework has always been in active development and has been widely endorsed by the cybersecurity community members. The main ATT&CK behavioral categories of malicious behaviors are recon, develop resources, acquire initial access, execute, persist, escalate privilege, evade defense systems, acquire credential access, discover, move laterally, collect, command and control, exfiltrate, and cause impacts [56].

Cybonto choose MITRE’s Structured Threat Information eXpression (STIX) to describe Asset subclasses and malicious campaigns under Group Activity. STIX subclasses are STIX Tools, STIX Malware, STIX Vulnerability, Cybox, and STIX Campaign [57]. STIX Tools describe legitimate software tools that can be leveraged by malicious actors to perform attacks. STIX Malware describes malicious programs that can compromise the confidentiality, integrity, or availability of the victims’ data. STIX Vulnerability describes vulnerabilities in legitimate software programs that can be exploited by malicious actors. Cybox—Cyber Observable eXpression—is a standardized language for describing cyber observables such as accounts, files, disks, devices, sessions, etc. STIX Campaign falls under the Group Activity subclass and describes specific sets of malicious behaviors that involve specific sets of targets, periods, and goals.

The use of “Group,” “Asset,” and their subclasses depends on each use case. For example, postarrest investigators may be only interested in Person and Asset classes to answer questions such as “Why did a hacker choose to attack a certain system and not others?” whereas threat intelligence teams may be interested in Person, Asset, Group, and other classes. In other words, usages of classes other than Person are nonconclusive and are subjected to inclusions or exclusions per each use case.

### Ranking Cybonto Core Constructs by Network Centrality Algorithms

**Figure 2:**
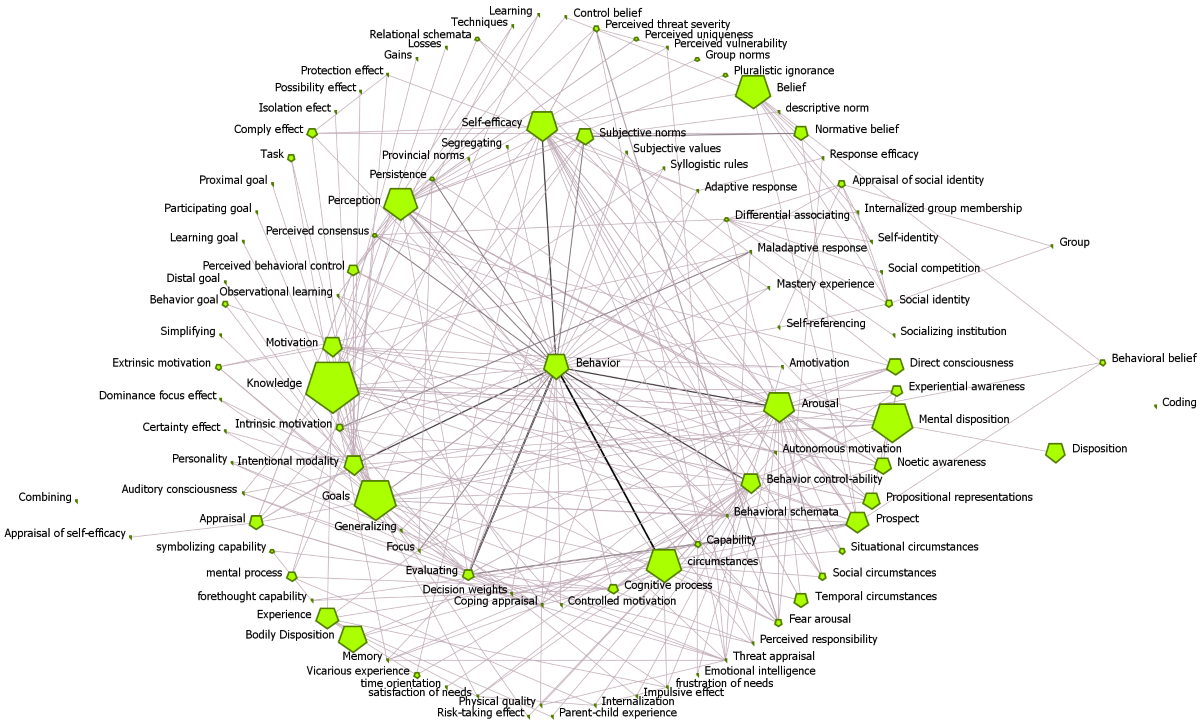
Cybonto "influence" relationships visualized.

Figure 2 shows the network of Cybonto core’s horizontal relationships. Constructs are nodes and the “influence” relationships are the edges. Each node’s size equals the log scale of the node’s page rank. A darker link color indicates a higher link value. Nodes were automatically arranged in a multi-circle layout with higher betweenness centrality (BC) nodes closer to the center. Key centrality metrics will be briefly described as follows.

Top authority centrality (AC) constructs receive influence from constructs that have the most influence on others. Top BC constructs are the ones that sit in the shortest paths among other constructs. BC constructs can serve either as bridges or gatekeepers of other constructs and processes. Top Eigenvector centrality (EC) constructs are the leaders of their cliques. A clique is a group of constructs in which each member has relationships with the others. In the context of the cognitive digital twin, a clique may represent a strong cognitive or behavioral pattern. Not only the top EC constructs are well-connected with their clique members, but also they also have relationships with other cliques.

Contribution centrality is EC on inverse-Jaccard weighted values of the input networks. A link between two constructs has the most contribution weight when the neighbors of one end are most different from the neighbors at the other end. Degree centrality (DC) has two submeasures—out-degree and in-degree. Top out-degree centrality constructs have the most out-links (influencing) to others while top incoming centrality constructs are influenced by the most important incoming neighbors. The top PageRank constructs have relationships with the most influential neighbors whether it is incoming or outgoing.

## Results

**Figure 3:**
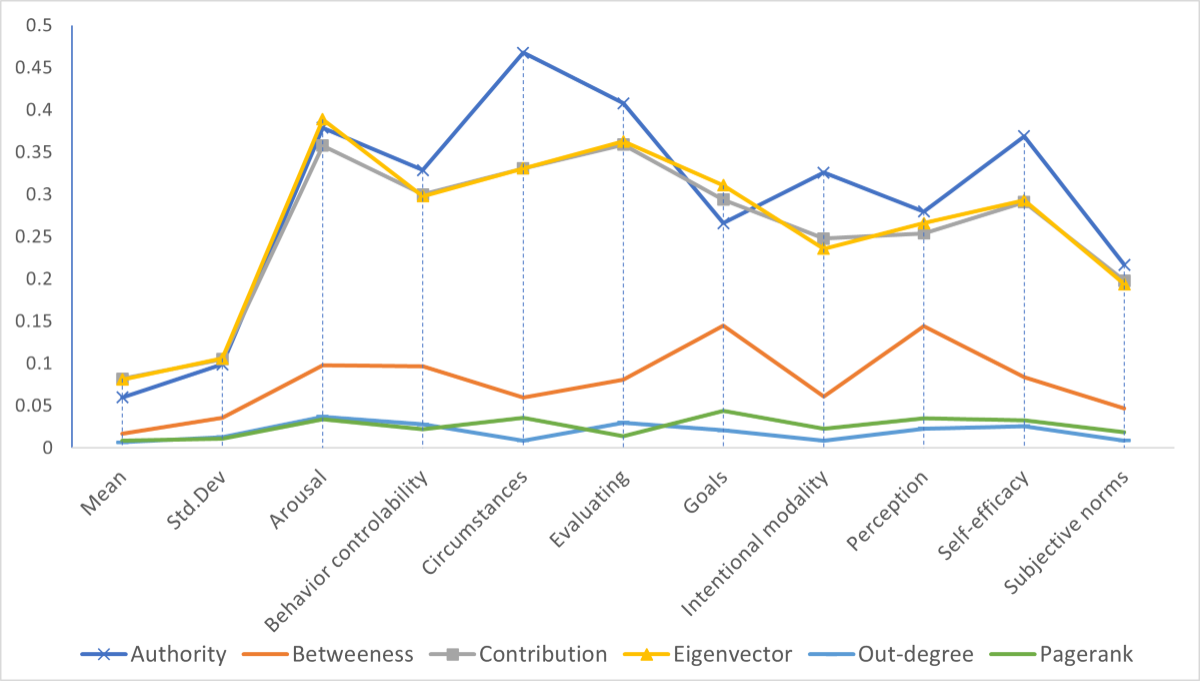
Most influential constructs.

The top 10 constructs across 20 network centrality measures are Behavior, Arousal, Goals, Perception, Self-efficacy, Circumstances, Evaluating, Behavior-Controllability, Knowledge, and Intentional Modality. Figure 3 shows the most influential constructs based on 6 different network centrality scores.

Table 2 presents top constructs’ specific fitted scores for 4 centrality categories. Depending on which centrality scores were chosen, there are differences in the ranking of constructs as is observable by comparing results in Figure 3 and Table 2. However, the differences are light. For example, most of the top constructs listed in Figure 3 remain within the top 20 with different reasonable choices of centralities.

A comprehensive report with scores, unscaled scores, and statistics across twenty network centrality scores are available at Cybonto-1.0 GitHub repository [53].

Among the top 9 most influential constructs shown in Figure 3, only Behaviors, Goals, Perception, Evaluating, and Knowledge are parts of existing digital cognitive architectures, and in most cases, are not explicitly implemented. It is possible that before this study, influential cognitive structures have been studied per independent use-cases and thus could not collectively attract attention from conservative cognitive system designers. Now with a birds-eye view across 20 behavioral theories, these top 10 constructs deserve better attention.

Within cognitive architectures, we may consider implementing Goals, Knowledge, Perception, and Evaluating explicitly and with finer granularity. For example, Perception is more than short-lived sensory perception. Alice perceives Bob as a nice guy, and such perception may persist even when Bob is no longer there with Alice. Finer structures mean more symbolic labels or more nodes in the knowledge graph and may lead to improvements such as more diverse rule firing mechanisms and more explainable information decay.

Additionally, we should consider adding Arousal and Intentional Modality. Although Arousal is a noncognitive construct, it is ranked in second place and influences several cognitive constructs within the top 10, such as Evaluating and Intentional Modality. Unfortunately, the current state of research regarding Arousal as a part of a digital cognitive process is almost nonexistent. SOAR-related research results show a few papers studying the effects of general emotions. ACT-R research repository shows just 4 papers studying the effects of Arousal on memory management.

Circumstance is another noncognitive construct with a significant influence on behavioral outcomes. The paper recommends expanding the existing Mental Image module in existing cognitive architectures to include nonphysical environment variables such as urgency, group dynamics, and social sentiments. Finally, the paper recommends a new component—Imagining—to enable the HDT to run its own situational simulations and reason about possible circumstances.

**Table 2:** Top constructs and their fitted key scores.

Constructs	Fit PR^a^	Fit EC^b^	Fit BC^c^	Fit DC^d^	Total
Behavior	10	10	10	5.333333	35.33333
Self-efficacy	2.978651	4.09735	5.791371	10	22.86737
Arousal	2.45894	6.494922	3.033944	8	19.98781
Goals	2.095989	4.048915	3.31916	6.666667	16.13073
Prospect	1.609572	2.008954	3.335824	8.666667	15.62102
Evaluating	3.373531	5.205153	2.811666	4	15.39035
Circumstances	2.225146	2.591971	2.975886	6.666667	14.45967
Behavior controllability	1.079106	1.051652	2.320296	6.666667	11.11772
Differential associating	1.938038	1.952155	4.191495	2.666667	10.74835
Knowledge	0.971335	3.448437	0.799434	5.333333	10.55254
Perception	1.933234	2.995944	1.233271	4	10.16245
Protection effect	3.419006	0.956777	1.811712	2	8.187495
Noetic awareness	0.800599	2.70121	0.248913	3.333333	7.084055
Intentional modality	0.948893	1.585625	0.357986	4	6.892503
Behavioral schemata	1.354209	4.679314	0.091006	0.666667	6.791195
Propositional representations	0.70164	2.70121	0.04671	3.333333	6.782894
Satisfaction of needs	0.381798	1.190226	1.13073	4	6.702753
Cognitive process	1.554735	2.509832	0.514903	1.333333	5.912803
Persistence	0.647449	2.104271	0.172818	2.666667	5.591204

^a^Fitted page rank.

^b^Fitted Eigenvector centrality.

^c^Fitted betweenness centrality.

^d^Fitted degree centrality.

## Discussion

### Principal Findings

Out of 108 psychology constructs, the top 10 are Behavior, Arousal, Goals, Perception, Self-efficacy, Circumstances, Evaluating, Behavior-Controllability, Knowledge, and Intentional Modality. In this list, only Behaviors, Goals, Perception, Evaluating, and Knowledge are parts of existing digital cognitive architectures. Notably, some of the constructs are not explicitly implemented. Early usability tests also demonstrate that Cybonto can be useful in other immediate uses such as manual analysis of hackers’ behaviors and automatic analysis of behavioral-cybersecurity knowledge texts.

### Usability Testing

#### Manual Analysis of Hackers’ Behaviors

The main goal of Cybonto is to provide one more reason for pushing current cognitive system designs, which may appear distant to some audience. Hence, this paper aims to demonstrate that Cybonto can be immediately employed in current cybersecurity-related tasks. Manual analysis of malicious actors’ behaviors is one essential task for cybersecurity intelligence gathering. It is also the first step in designing a virtual human digital twin of a real hacker. The demonstration is as follows.

A small group of cybersecurity professionals working in one of the US Federal Reserve Bank’s branches participated in a Cybonto workshop. Group members had to choose either Snowden’s biography or Pavlovich’s biography as their reading assignment before the workshop. Both Snowden and Pavlovich are notorious cyber actors. In the workshop, participants were taught a simplified version of Cybonto. Notably, most of the members do not have a background in behavioral psychology. A table with columns of Knowledge, Expectation, Attitudes, Behavioral Belief, Normative Belief, Control belief, Intents, Subjective Norms, Perceived Behavioral Control, Actual Behavioral Control, Social Involvements, Social Attachment, and Social Commitment was provided. The goal was to have members establish a basic behavioral profile for each actor by filling values ranging from 0 to 6 in each of the table’s columns.

**Figure 4:**
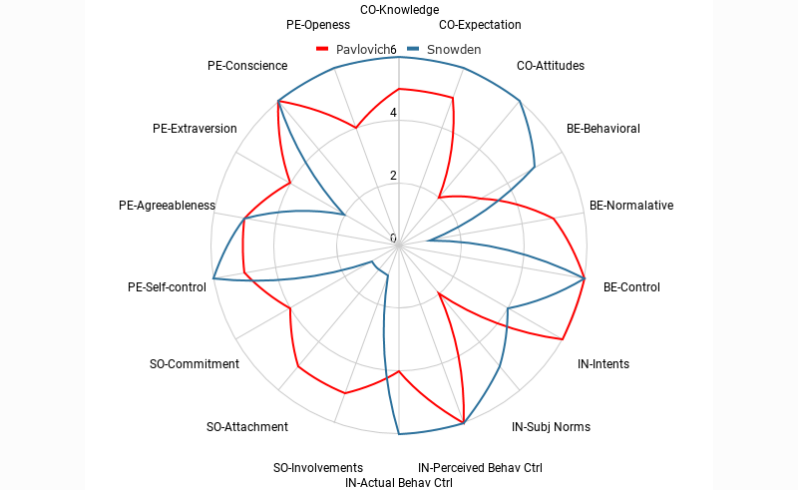
Behavioral differences between Snowden and Pavlovich. BE: belief; CO: cognitive; Ctrl: control; IN: intentions; SO: social bonds; PE: personality.

Members of the group who read Snowden’s biography book (the Snowden group) presented evidence for each column. The strength of evidence would determine the relevant column’s score. Members in the other group (the Pavlovich group) may debate about the Snowden group’s analysis and scoring. In the case of a stalemate, the author would assist with negotiating the scores. The same process was used for establishing Pavlovich’s behavioral profile. The workshop lasted 2 hours and produced results shown in Figure 4.

Overall, this usability test has shown that (1) Cybonto can be friendly to the professionals who do not have a behavioral psychology background; (2) Cybonto is descriptive and can help with pointing out the behavioral differences between two distinct cyber actors; (3) Cybonto is consistent so that consensus in a manual analysis of cyber actors can be reached within a reasonable amount of time.

#### Analysis of Behavioral Cybersecurity Research Papers

Cybonto can also be used in machine learning–assisted domain knowledge analysis. For a demonstration, more than 3000 full texts of behavioral cybersecurity research within the past 5 years were downloaded from Google Scholar. A total of 2380 PDF files were selected and converted to plain text files. Natural language processing techniques were deployed on the text files and produced a concept list. The automatically generated list was then manually inspected and mapped into corresponding Cybonto constructs. A meta-network of related Cybonto’s constructs in each document was generated. Then, analysis was carried out on a unionized meta-network of all document-level meta-networks.

**Figure 5:**
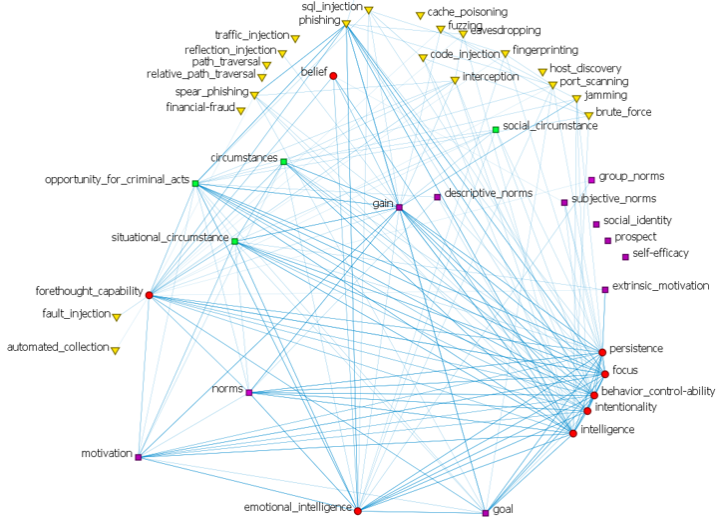
Analysis snapshot of behavioral cybersecurity research papers within the past 5 years.

Figure 5 provides a snapshot of the result with the following observations. Gain appears to be the most discussed construct with healthy connections to construct groups of attacks (yellow triangles), circumstances (green squares), and personality (red dots). Most studies focused on the direct relationships between the attacks and hackers’ personalities. A much lower number of studies focused on how circumstances may directly influence malicious behaviors.

Overall, this simple experiment shows that Cybonto can be used to automatically analyze texts within the intersection of behavioral psychology and cybersecurity. Analyzed results may provide insights such as knowledge gaps and imbalance. Such interdisciplinary capabilities can be beneficial to teams with limited expertise. Future general artificial intelligence agents may also leverage Cybonto for their automatic knowledge analysis and acquisition.

### Expanding the Vision With The Cybonto Conceptual Framework

The novel Cybonto conceptual framework aims to provide general directions on answering the previously mentioned questions regarding the vision of DTs and HDTs for cybersecurity. The framework targets the cognitive process of a malicious actor as an HDT within a DT system. Cognitive space is defined by the behavioral or cognitive component of the Cybonto ontology. The action space is limited by the HDT's set of encoded actions, its ability to improvise new moves, and the other DTs’ interaction interfaces.

**Figure 6:**
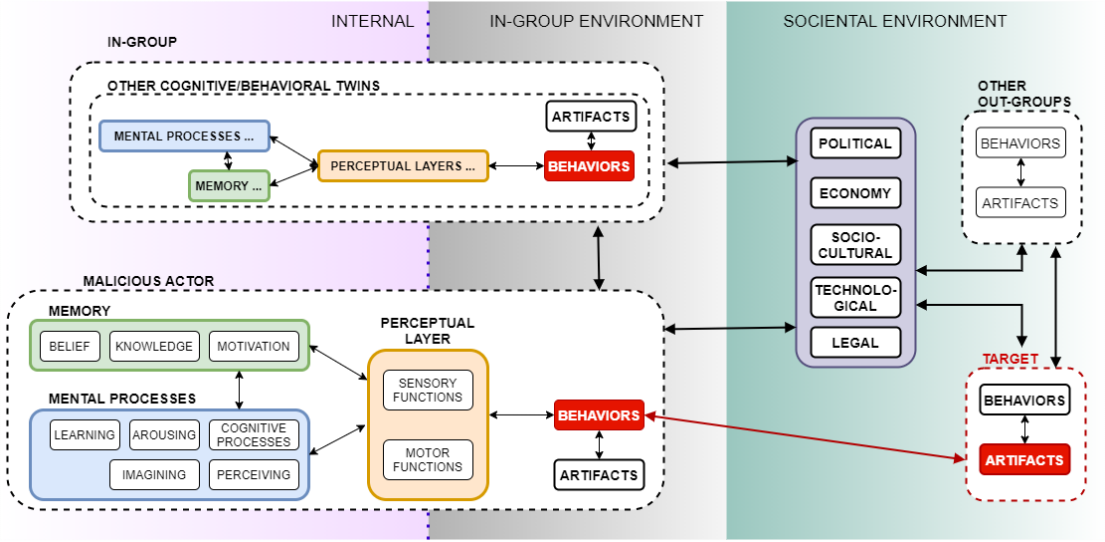
The Cybonto conceptual framework.

The Cybonto conceptual framework was formed upon analysis of the Cybonto ontology. Figure 6 presents the Cybonto conceptual framework with 3 environment types and four groups of digital twins.

The internal environment (INE) is private to each DT. It contains both cognitive components and noncognitive components. Opposite to the internal environment is the societal environment (SOE) where everything is public. In between, the in-group environment (IGE) connects INE with SOE. All environments follow the Bronfenbrenner Ecological System Theory [58], which describes influences as progressive, varying, and reciprocal forces among individuals and environments. For example, a seemingly distant public event may still be able to affect certain private mental processes.

The IGE and the SOE are relative to the malicious HDT. The IGE is equivalent to the Bronfenbrenner Micro and Meso systems. The microsystem is the most inﬂuential external environment with members such as family, close friends, school, lovers, and mentors. SOE is equivalent to the Bronfenbrenner Exo, Macro, and Chrono systems. The Cybonto conceptual framework requires four representatives from 4 DT groups. We need one attacker HDT and one defender HDT. Unlike traditional models to which data and feature specifications were explicitly fed, an attacker HDT must collect the data by itself. Group-related data cannot be inferred if the fundamental group structure is not met. Hence, we then need at least two more DTs to present IGE and SOE identities.

An HDT can perform two main types of behaviors: the artifact-creating or -altering behavior and the nonartifact behavior. An artifact can range from a piece of code to a complex noncognitive digital twin. Viewing a malware’s codes is a nonartifact behavior, while running the codes can be an artifact-altering behavior if the codes make changes to other artifacts. The perceptual layer sits on the border between the internal and external environments (IGE and SOE). Different perceptual layers in combination with different cognitive systems will have different perceptions of the same data streams. Refined perceptions constitute only a small part of a digital cognitive system. The Cybonto ontology details thousands of cognitive paths for processing initial perceptions. The result of a cognitive processing chain will be either a nonartifact behavior or an artifact-creating or -altering behavior. The behaviors (data streams) will be observed by other HDTs, and a new round of feedback loops begins. It is essential to note that a behavior can be kept secret within the in-group environment.

In this framework, (1) HDTs have the complete freedom to interact with other DTs per published protocols, and automatically seek whatever data are made available to them. (2) By releasing their behaviors, HDTs generate new data, which may then be consumed by other HDTs. (3) The cognitive architecture within each HDT determines its cognitive capabilities, which should include awareness and adaptation. (4) Cybonto DT simulation’s objectives should be more about discovering new knowledge (the *why* and *how*) rather than mining specific data (the *what*).

### Limitations

The biggest internal threat to validity is the maturation of the Cybonto ontology. The current Cybonto version should be treated as the “alpha release,” and numerous development steps will be needed. First, the mapping of each theory to triplets of (construct, influence, and construct) must be cross-checked by more psychologists. Second, missing and duplicated constructs must be identified by careful vetting and deliberations. Finally, ontology testing steps must be carried out. The risk of bias theory selection should be minimal as more theories will be incorporated over time.

The biggest external threat to validity is the various implementations of Cybonto. Understandably, solution developers should only implement the Cybonto constructs that are needed for solving their practical problems. In other cases, solution developers must add new constructs that were not packaged with Cybonto. Uncareful addition and removal of constructs may weaken Cybonto integrity leading to faulty performance. Additionally, certain feedback loops must exist for certain psychology or cognitive paths to “fire.” For instance, an HDT may need to gather enough information about a situation from other HDTs and DTs before it can reason about the situation. Hopefully, the proposed Cybonto Conceptual Framework will help with minimizing these external threats to validity.

### Prior Work

Booker and Musman [59] indicated that human-in-the-loop cybersecurity responses are slow because cyberattacks happen at a higher speed than human decision-making. Therefore, we need autonomous agents of which behaviors are aligned with the defenders’ understanding of related business aspects and preferences. The author framed the problem as a partially observable Markov decision problem, in which “Belief” is the probability of being in a particular state, provided the agents know some past actions and observations. Without using a cognitive system, the work demonstrates the usefulness of autonomous agents for the task of finding out good defense strategies under developing attacks.

According to Francia et al [60], predicting the outcomes of risky behaviors in cyberspace is challenging owing to sensitivity to initial conditions, occurrences of random events, and interactivity among different complex systems. The paper proposed agent-based modeling of entity behavior in cybersecurity as one solution. The study simulated different scenarios of computer virus spread. Simulation parameters are the sophistication of hackers’ attacks, trust level, defenders’ level of training, and quality of cyber defense. Although the study is a work in progress, it demonstrates the mechanisms and the benefits of having opposed autonomous agents interact with each other. From another angle, Metge et al [61] investigated the dynamic trust relationships among autonomous agents and human operators who are all on the same team. The paper emphasized the challenge of building the right autonomous agent’s mental model, which is the first step in gaining human operators’ trust. Autonomous agents need to be both able to provide sound solutions and to behave in ways that their human counterparts can trust.

Thomson et al [62] proposed ACT-R–based models as autonomous cybersecurity agents that can understand and augment human analysts. Interestingly, digital agents can detect bias in human teammates. The paper describes in adequate detail the working of ACT-R in 3 use cases of making sense of human decisions, cyber-deceptive signaling defense, and malware detection. In another study, Golovianko et al [63] used Pi-Mind and adversarial machine learning to clone image classification cognitive capabilities of human participants. The study also reviewed important concepts such as top-down cognitive twin cloning via explicit transfer of knowledge, bottom-up cloning via machine learning or deep learning, and individual and group cloning. Notably, the study considers autonomous agents as “cognitive clones” or “cognitive twins,” all of which can act like the human counterparts in both business-as-usual situations and critical situations. The results illustrate more stable performances of cognitive twins in stressful situations.

### Conclusions

DCTs and HDTs are gaining popularity, but they are not necessarily new concepts. A good body of prior works involves “autonomous agents” with various applications in security and cybersecurity. However, autonomous agents have been designed in specific ways for solving specific problems. HDTs are fundamentally different from autonomous agents. Most HDTs consist of a cognitive system and a noncognitive system, and most cognitive systems can combine cognitive reasoning (symbolic) with deep learning models (subsymbolic). Furthermore, HDTs and DTCs should be able to perform in a much wider set of situations than autonomous agents as DCTs are parts of HDTs that are in turn a part of the Metaverse strategy. Once massive noncognitive digital twin systems transition to the internet, adding human cognitive digital twins will be the only logical next step.

The vision of letting human digital twins ”run free“ in connected digital twin worlds (the Metaverse) and observing them is realistic and offers a new paradigm in knowledge mining. The Cybonto conceptual framework demonstrates how such an ecosystem can be leveraged for shaping proactive cybersecurity defense strategies. In the context of studying malicious cybersecurity behaviors, the key is building a digital human cognitive twin that models well malicious hackers' cognitive patterns. Specifically, cognitive reasoning with adequate granularity and a well-designed ontology allows us to observe, understand, and—more importantly—explain the HDTs’ behaviors. Hence, the paper also proposes the Cybonto ontology as a recommendation on how current cognitive systems can be extended.

Notably, medical researchers may take Cybonto core ontology and fit it to their applications such as virtual patients for applied psychology training, automatic behavioral annotations, analysis of electronic health records, and virtual agents for community psychology experiments. Future work may involve further framework development, fine-tuning and expanding the ontology, human cognitive cloning, and building different practical HDTs.

## Abbreviations

AC: authority centrality
BC: betweenness centrality
BFO: Basic Formal Ontology
DC: degree centrality
DCT: Digital Cognitive Twin
DT: Digital Twin
EC: Eigenvector centrality
HDT: Human Digital Twin
IGE: in-group environment
INE: internal environment
MF: Mental Functioning
SOE: societal environment
STIX: Structured Threat Information eXpression
